# Supplementary material for: The p63 C-terminus is essential for murine oocyte integrity
Source: Nat Commun. 2021 Jan 15;12:383. doi: 10.1038/s41467-020-20669-0 (PMC7810856; doi:10.1038/s41467-020-20669-0)
Supplement: Supplementary file 1 — Supplementary Information [file 41467_2020_20669_MOESM1_ESM.pdf]

Supplementary Information

The p63 C-terminus is essential for murine oocytes integrity

Lena et al

Supplementary Table 1 PCR Primers	
m3'UTR-R	TGTAGGGGCTGGGAGGTGGAAG
mEX13F	GACCACCATCTATCAGATTGAGC
mEX12F	GACTCAGCCCTACCCAAGCTCTC
mEX11F	GATGCAGTCTCAGTCTTCATATG
mEX10F	GTGAGAGGTCGTGAGACGTAC
mEX10'R	CTATGGGTACACGGAGTGGTT
m-bactinF	TGTCCCTGTATGCCTCTGGTCG
m-bactinR	GAACCGCTCGTTGCCAATAGTG
mJag2F	CAAGTTCTGTGACGAGTGTGTCCC
mJag2R	TTGCCCAAGTAGCCATCTGG
mJag1F	CAAAGTGTGCCTCAAGGAGTATCAG
mJag1R	TCCACCAGCAAAGTGTAGGACCTC
mFgfR2-IIIbF	CCCATCCTCCAAGCTGGACTGCCT
mFgfR2-IIIbR	CAGAACTGTCAACCATGCAGAGTG
mK14F	TCCCAATTCTCCTCATCCTC
mK14R	TAGTTCTTGGTGCGCAGGAC
mCdkn1aF	TGTTCCGCACAGGAGCAAAGTG
mCdkn1aR	CGAAGTCAAAGTTCCACCGTTCTCG
mPumaF	AGGGAAGGGAGGGCTGAAGG
mPumaR	GAGGCCAGGCCCAAAGTGAA
mNoxaF	CGCTGGTGCTGCCTACTGAA
mNoxaR	GCCTTTCTCCCGGGCATCTC
For genot	GCACTCCTTGGACCTCAGC
Rev genot	TGTGTTGCCCAATCAGGTCTC

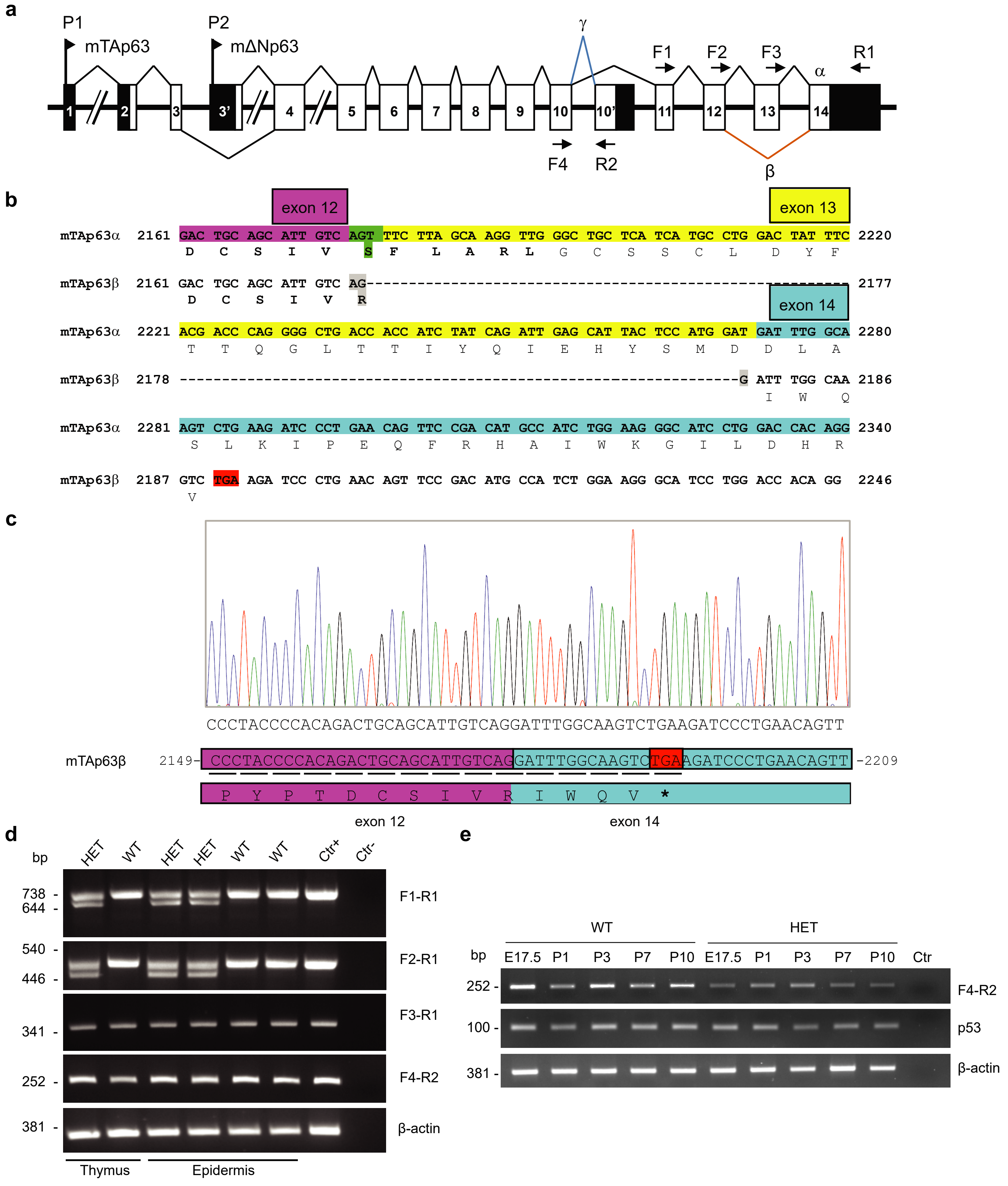

Supplementary Figure 1. Expression of p63 isoform in Δ13p63 heterozygous mice. (a) *Trp63* gene structure. The semi-quantitative PCR primers F1, F2, F3, F4, R1 and R2 are depicted; **(b)** mTAp63α and TAp63β isoform nucleotide and amino acid alignments. The exons and different amino acids are indicated by different colours; **(c)** Sequencing reaction electropherogram of the agarose gel-purified PCR fragments obtained by amplifying the Δ13p63 cDNA allele expressed in thymus of new-born HET mice; **(d)** Semi-quantitative PCR analysis of p63 isoforms expression in P1 epidermis and thymus of WT and HET Δ13p63 (indicated as HET in figure) mice. The PCR primers are described in (a). Ctr+ indicates cDNA obtained from reverse transcription of the total RNA extracted by WT mouse keratinocytes; Ctr- indicates a no-template PCR; **(e)** Semi-quantitative PCR analysis of p63γ isoforms and p53 expression in ovaries isolated at stages E17.5, P1, P3, P7, P10 of WT and HET mice. PCR primers are described in (a). Ctr- indicates a no-template PCR. Source data are provided as a Source Data file.

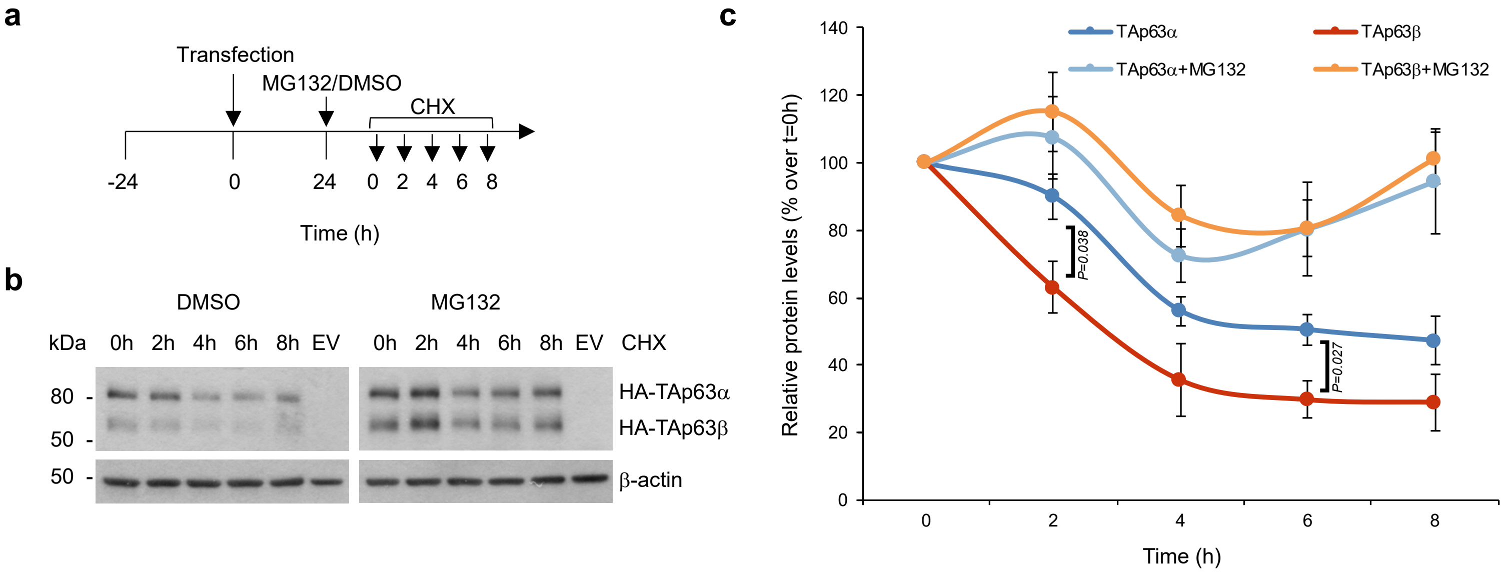

Supplementary Figure 2. TAp63β isoform degrades faster than TAp63α. (**a**) H1299 cells were co- transfected with HA-tagged TAp63α and TAp63β expression vectors (1:1 ratio); 24h after transfection cells were treated with the proteasome inhibitor MG132, or DMSO as control, and one hour later with cycloheximide (CHX). Cells were collected after 0, 2, 4, 6, 8h; (**b**) Protein levels detection by western blot at 0, 2, 4, 6, 8h after CHX treatment using an anti-HA antibody. β-actin was used as loading control and for normalization in the densitometric analysis; one representative experiment of four is shown; (**c**) Densitometric quantification of TAp63α and TAp63β protein levels at the collected time points. n=4 biologically independent experiments. Data are presented as mean ± SEM, p-value by two-tailed unpaired Student's *t*-test, no adjustments for multiple comparison were made. Source data are provided as a Source Data file.

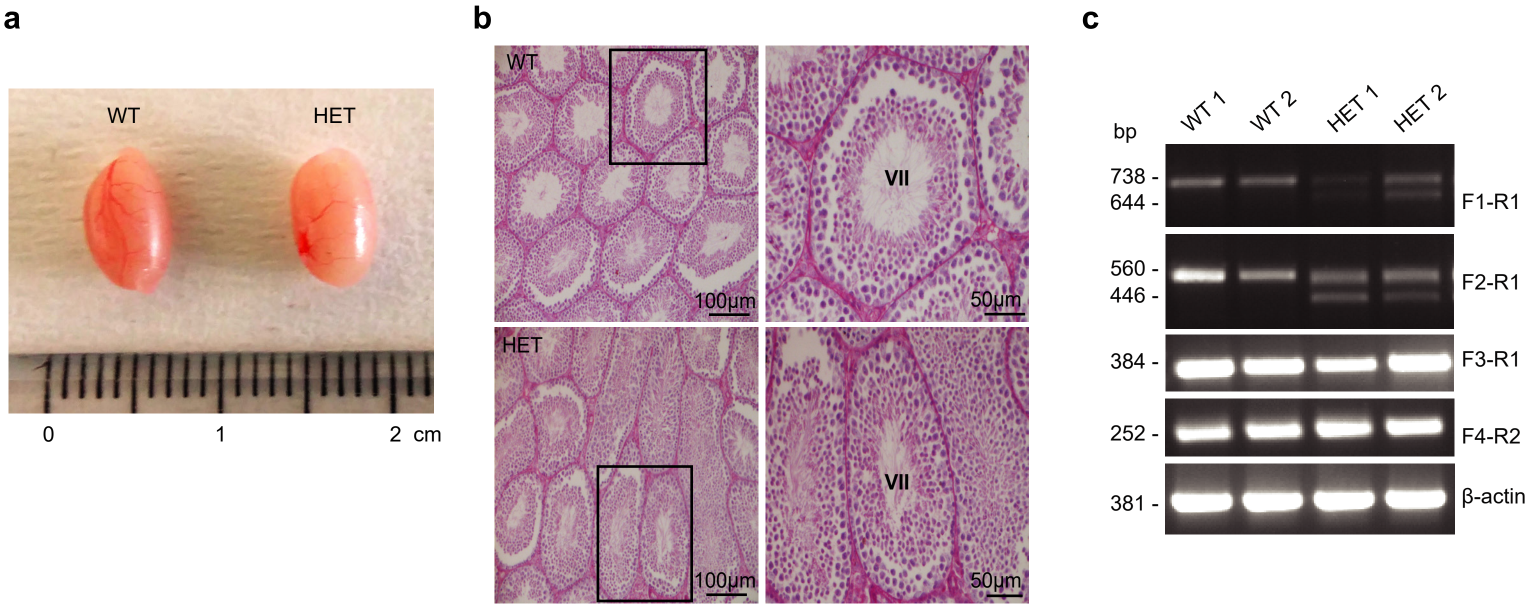

Supplementary Figure 3. Characterization Δ13p63 heterozygous testis. (**a**) P45 WT and HET Δ13p63 (indicated as HET in figure) testis size; (**b**) PAS staining of adult (P45) testis sections of WT and HET mice. The panels on the right side are magnifications of the areas in the black boxes. Both WT and HET testes showed normal appearances with round and elongate spermatids in stage VII tubules; (**c**) Semi-quantitative PCR analysis of p63α and p63β isoforms expression at P45 testes of WT and HET (n=2) mice; β-actin was used as a housekeeping gene for normalization. The PCR primers are described in Supplementary Figure 1a. Source data are provided as a Source Data file.

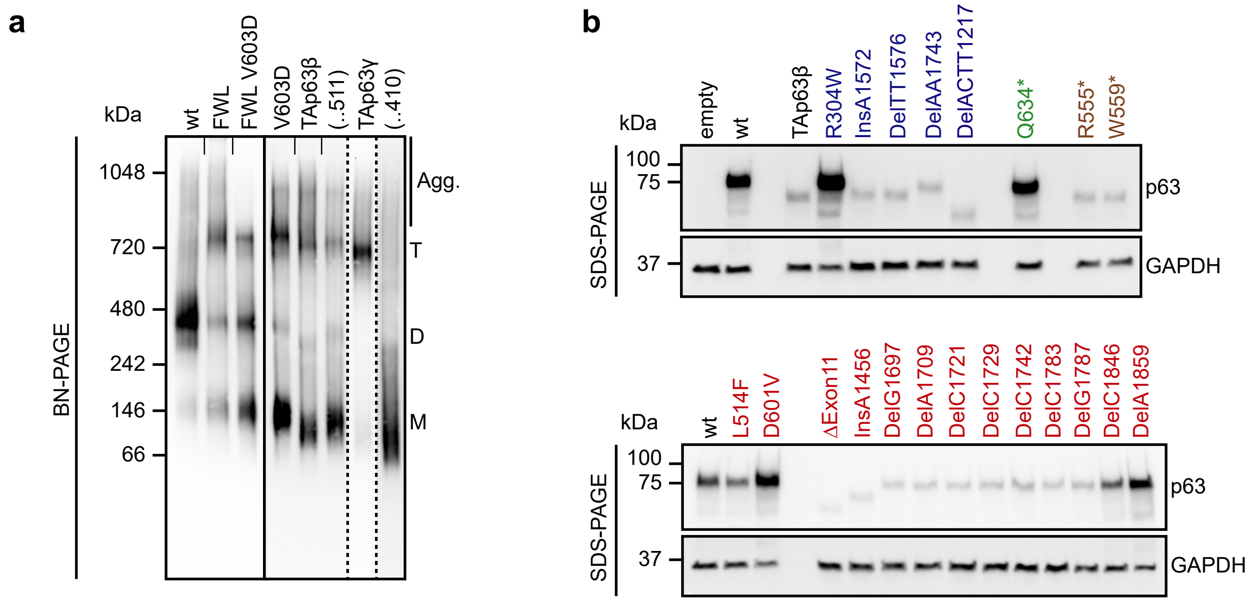

Supplementary Figure 4. **WB analysis of TAp63 isoform and mutant expression in luciferase assay extracts.** (**a**) Oligomeric state analysis of the indicated p63 isoforms and C-terminal truncations via BN- PAGE expressed transiently in transfected H1299 cells. The oligomeric conformation is indicated by T (tetramer), D (dimer), M (monomer) and, additionally, Agg. for aggregation; (**b**) WB analysis of mutant TAp63α expression in the luciferase assay extracts described in Figure 5b. GAPDH was used as loading control. Source data are provided as a Source Data file.

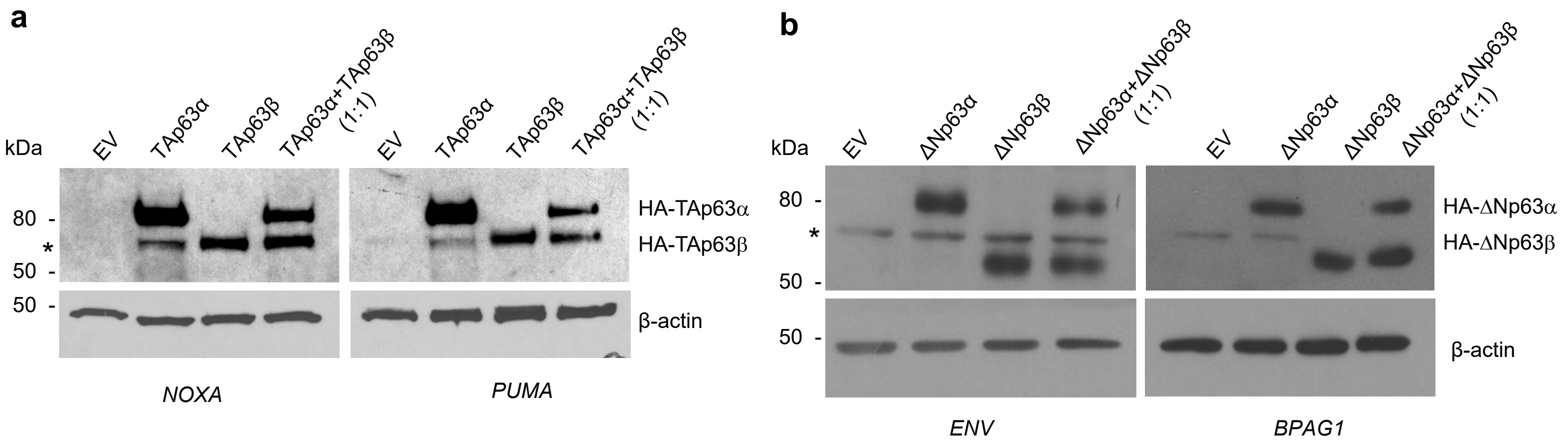

Supplementary Figure 5. **WB analysis of TAp63 and ΔNp63 isoforms expression in luciferase assay extracts.** (**a**) WB analysis of TAp63 isoforms expression in the luciferase assay extracts described in Figure 4d. β-Actin was used as a loading control. *, non-specific band; (**b**) WB analysis of ΔNp63 isoforms expression in the luciferase assay extracts described in Figure 4e; β-actin was used as a loading control. *, non-specific band. Source data are provided as a Source Data file.

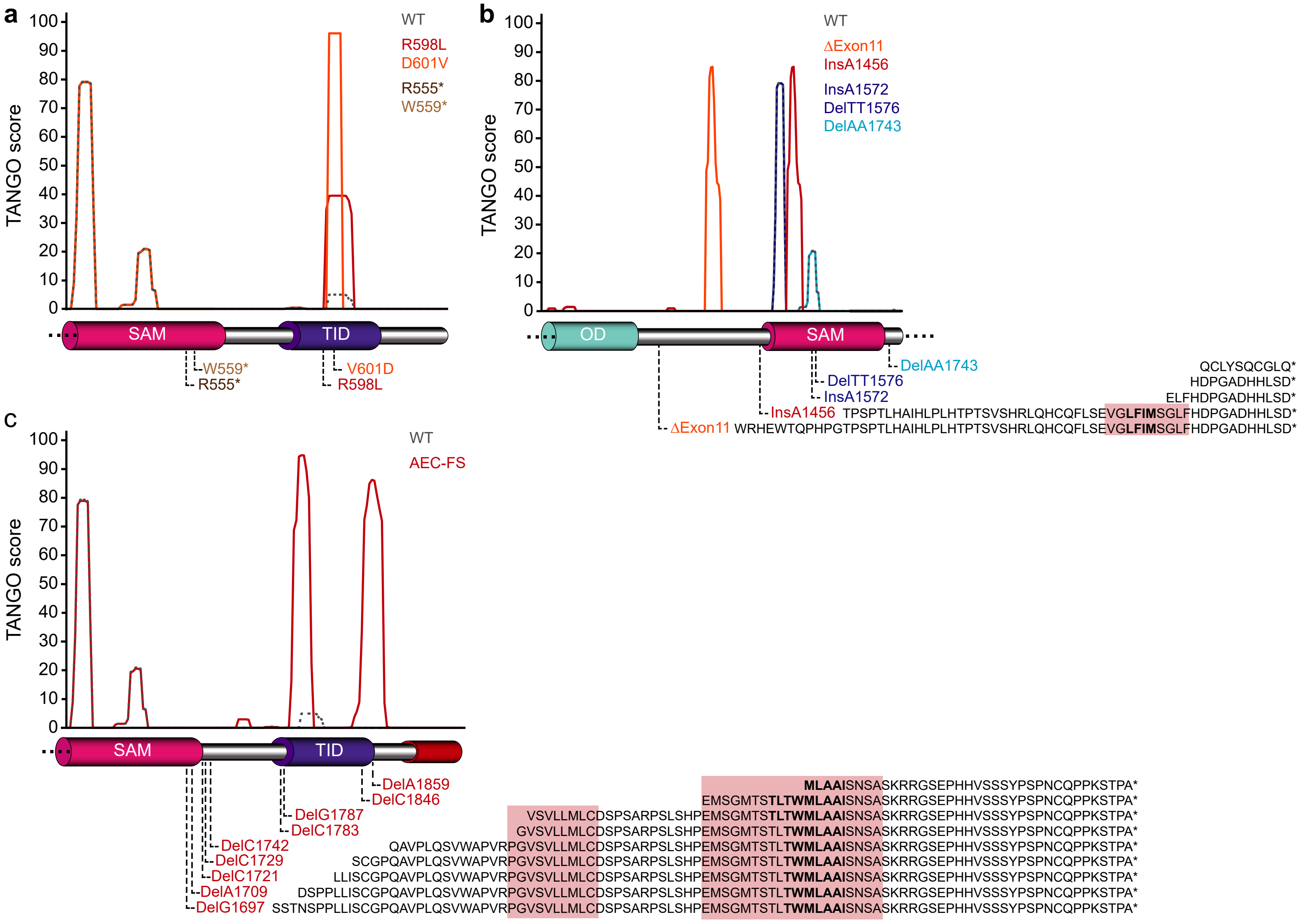

d

p63 mutations	Deletion or mutation of aggregation-prone regions (APRs)	
R555* W559*	Δ508-514 Δ531-540	ΔAPRs
	V511D T533D	mutAPRs
ΔExon11 InsA1456	Δ517-526	ΔAPR
DelA1709	Δ596-603 Δ624-631	ΔAPRs
DelA1859	Δ624-631	ΔAPR

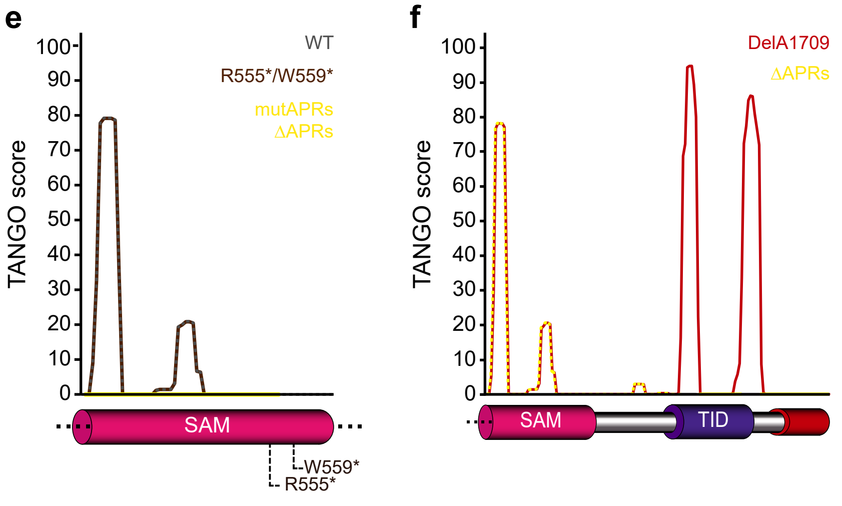

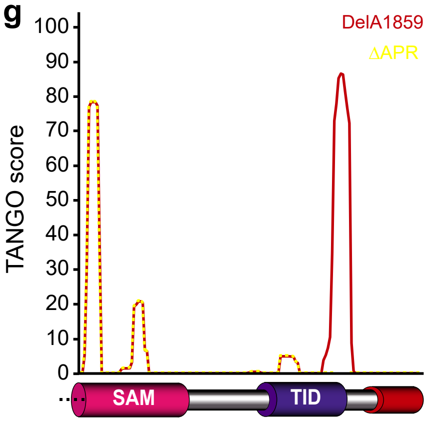

Supplementary Fig 6. *In silico* analysis of aggregation prone regions by TANGO algorithm. Analysis was performed using the following parameters: pH 7.2, 4°C, ionic strength 150 mM. (**a-c**) TANGO prediction of aggregation prone peptides for AEC/RHS, POI and ELA mutations compared to WT p63α; (**d**) POI and selected AEC/RHS mutant p63α aggregation rescue mutations by either deletion of the aggregation prone region (APR) or Asp mutation, named in the following uniformly ΔAPR or mutAPR; (**e-g**) TANGO predictions of selected mutant p63α aggregation rescue mutations, ΔAPR or mutAPR, compared to mutant p63α.

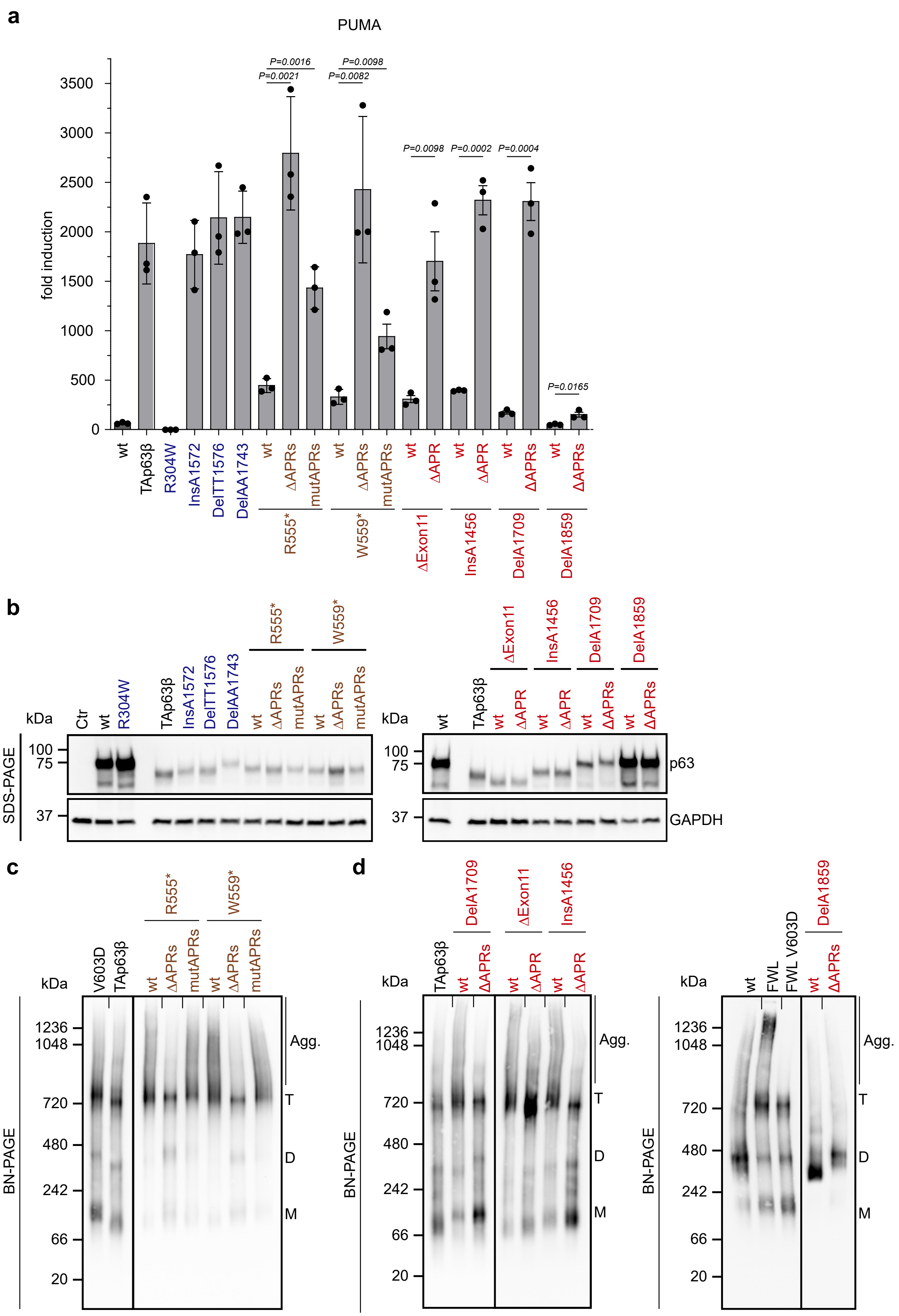

Supplementary Fig 7. POI mutations (R555*, W559*) show an aggregation mechanism similar to AEC/RHS syndrome. (**a**) Reporter luciferase assay of syndromic, mutant TAp63α and rescue mutations by deletion of APR (ΔAPR) or Asp mutations (mutAPR). n=3 biologically independent experiments, luciferase assay was performed in triplicates on *PUMA* 4xBS2WT responsive elements (REs). Data are presented as mean ± SD, p-value by two-tailed unpaired Student's *t*-test, no adjustments for multiple comparison were made; (**b**) WB analysis of mutant TAp63α ΔAPR or mutAPR expression in the luciferase assay extracts described in a). GAPDH was used as loading control (**c-d**) BN-PAGE analyzation of mutant TAp63α and rescue mutations. Aggregation is decreased in by deletion of predicted aggregation prone regions (ΔAPR). The oligomeric conformation is indicated by T (tetramer), D (dimer), M (monomer) and, additionally, Agg. for aggregation. Source data are provided as a Source Data file.
