# Supplementary material for: The p63 C-terminus is essential for murine oocyte integrity
Source: Nat Commun. 2021 Jan 15;12:383. doi: 10.1038/s41467-020-20669-0 (PMC7810856; doi:10.1038/s41467-020-20669-0)
Supplement: Supplementary file 2 — Reporting Summary [file 41467_2020_20669_MOESM2_ESM.pdf]

nature research

Corresponding author(s): Eleonora CANDI

Last updated by author(s): 24/11/2020

Reporting Summary

Nature Research wishes to improve the reproducibility of the work that we publish. This form provides structure for consistency and transparency in reporting. For further information on Nature Research policies, see Authors & Referees and the Editorial Policy Checklist .

Statistics

For all statistical analyses, confirm that the following items are present in the figure legend, table legend, main text, or Methods section.

n/a	Confirmed	
☐	☒	The exact sample size (*n*) for each experimental group/condition, given as a discrete number and unit of measurement
☐	☒	A statement on whether measurements were taken from distinct samples or whether the same sample was measured repeatedly
☐	☒	The statistical test(s) used AND whether they are one- or two-sided<br>*Only common tests should be described solely by name; describe more complex techniques in the Methods section.*
☒	☐	A description of all covariates tested
☐	☒	A description of any assumptions or corrections, such as tests of normality and adjustment for multiple comparisons
☐	☒	A full description of the statistical parameters including central tendency (e.g. means) or other basic estimates (e.g. regression coefficient) AND variation (e.g. standard deviation) or associated estimates of uncertainty (e.g. confidence intervals)
☒	☐	For null hypothesis testing, the test statistic (e.g. *F*, *t*, *r*) with confidence intervals, effect sizes, degrees of freedom and *P* value noted<br>*Give P values as exact values whenever suitable.*
☒	☐	For Bayesian analysis, information on the choice of priors and Markov chain Monte Carlo settings
☒	☐	For hierarchical and complex designs, identification of the appropriate level for tests and full reporting of outcomes
☒	☐	Estimates of effect sizes (e.g. Cohen's *d*, Pearson's *r*), indicating how they were calculated

Our web collection on statistics for biologists contains articles on many of the points above.

Software and code

Policy information about availability of computer code

Data collection	NIS Elements software (Nikon)<br>Applied Biosystems 7500 Real-Time PCR Software (ThermoFisher)
Data analysis	NIS Elements software (Nikon, Tokyo, Japan)<br>Applied Biosystems 7500 Real-Time PCR Software (ThermoFisher)<br>TANGO algorithm (http://tango.crg.es/)<br>GraphPad software

For manuscripts utilizing custom algorithms or software that are central to the research but not yet described in published literature, software must be made available to editors/reviewers. We strongly encourage code deposition in a community repository (e.g. GitHub). See the Nature Research guidelines for submitting code & software for further information.

Data

Policy information about availability of data

All manuscripts must include a data availability statement. This statement should provide the following information, where applicable:

- Accession codes, unique identifiers, or web links for publicly available datasets
- A list of figures that have associated raw data
- A description of any restrictions on data availability

The data that support the findings of this study are available from the corresponding author upon reasonable request.

Field-specific reporting

Please select the one below that is the best fit for your research. If you are not sure, read the appropriate sections before making your selection.

- [x] Life sciences
- [ ] Behavioural & social sciences
- [ ] Ecological, evolutionary & environmental sciences

For a reference copy of the document with all sections, see nature.com/documents/nr-reporting-summary-flat.pdf

Life sciences study design

All studies must disclose on these points even when the disclosure is negative.

Sample size	No statistical methods were used to determine the sample size. The sample size was sufficient to yield high statistical significance.
Data exclusions	No samples were excluded.
Replication	All experimental findings were reproduced.
Randomization	No randomization was performed.
Blinding	No blinding was used for the data collection or analysis.

Reporting for specific materials, systems and methods

We require information from authors about some types of materials, experimental systems and methods used in many studies. Here, indicate whether each material, system or method listed is relevant to your study. If you are not sure if a list item applies to your research, read the appropriate section before selecting a response.

Materials & experimental systems

n/a	Involved in the study	
[ ]	[x]	Antibodies
[ ]	[x]	Eukaryotic cell lines
[x]	[ ]	Palaeontology
[ ]	[x]	Animals and other organisms
[x]	[ ]	Human research participants
[x]	[ ]	Clinical data

Methods

n/a	Involved in the study	
[x]	[ ]	ChIP-seq
[x]	[ ]	Flow cytometry
[x]	[ ]	MRI-based neuroimaging

Antibodies

Antibodies used	All the antibodies used in this study are mentioned in the Material and Methods section.
Validation	The antibodies were validated by the manufacturer and/or in previously published articles.

Eukaryotic cell lines

Policy information about cell lines

Cell line source(s)	H1299 and HEK293 were purchased from ATCC.
Authentication	No authentication was performed.
Mycoplasma contamination	Cell lines were routinely tested for mycoplasma contamination using MycoAlert kit (Lonza cat no LT07-218).
Commonly misidentified lines (See ICLAC register)	No commonly misidentified cell lines were used.

Animals and other organisms

Policy information about studies involving animals; ARRIVE guidelines recommended for reporting animal research

Laboratory animals	Generation of new genetically-modified mice is described in Material and Methods section.<br>All mice had C57Bl/6J background.

Wild animals	No wild animals were used in this study.
Field-collected samples	No samples were collected from the field.
Ethics oversight	All experiments were approved by the Institutional Animal Care and Use Committee (IACUC) and were carried out according to the Italian and European rules (D.L.116/92; C.E. 609/86; European Directive 2010/63/EU). For mice experiments license n° is 817/2016PR (Italian Ministry of Health

Note that full information on the approval of the study protocol must also be provided in the manuscript.
